# Organic Aciduria Disorders in Pregnancy: An Overview of Metabolic Considerations

**DOI:** 10.3390/metabo13040518

**Published:** 2023-04-04

**Authors:** Loai A. Shakerdi, Barbara Gillman, Emma Corcoran, Jenny McNulty, Eileen P. Treacy

**Affiliations:** 1 National Centre for Inherited Metabolic Disorders, Mater Misericordiae University Hospital, D07 R2WY Dublin, Ireland; 2 National Centre for Inherited Metabolic Disorders (NCIMD), Childrens Health Ireland at Temple Street, Temple Street, D01 XD99 Dublin, Ireland; 3 The Irish National Rare Disease Office, Mater Misericordiae University Hospital, D07 R2WY Dublin, Ireland; 4 Discipline of Medicine, School of Medicine, Trinity College Dublin, D02 PN40 Dublin, Ireland; 5 University College Dublin (UCD) School of Medicine, Belfield, D04 V1W8 Dublin, Ireland

**Keywords:** inborn errors of metabolism, organic aciduria, pregnancy, postpartum

## Abstract

Organic acidurias are a heterogeneous group of rare inherited metabolic disorders (IMDs) caused by a deficiency of an enzyme or a transport protein involved in the intermediary metabolic pathways. These enzymatic defects lead to an accumulation of organic acids in different tissues and their subsequent excretion in urine. Organic acidurias include maple syrup urine disease, propionic aciduria, methylmalonic aciduria, isovaleric aciduria, and glutaric aciduria type 1. Clinical features vary between different organic acid disorders and may present with severe complications. An increasing number of women with rare IMDs are reporting successful pregnancy outcomes. Normal pregnancy causes profound anatomical, biochemical and physiological changes. Significant changes in metabolism and nutritional requirements take place during different stages of pregnancy in IMDs. Foetal demands increase with the progression of pregnancy, representing a challenging biological stressor in patients with organic acidurias as well as catabolic states post-delivery. In this work, we present an overview of metabolic considerations for pregnancy in patients with organic acidurias.

## 1. Introduction

Organic acidurias (synonym: organic acid disorders, organic acidemias, (OADs)) are a heterogeneous group of rare IMDs caused by the deficiency of an enzyme or a transport protein involved in the intermediary metabolic pathways. All known organic acidurias are inherited as autosomal recessive traits. There are now considered to be up to 1500 monogenic disorders affecting metabolism. A recent international classification, the International Classification of Inherited Metabolic Disorders (ICIMD described 24 categories. Thirteen groups consist of disorders of intermediary metabolism [1]. Category 1 includes disorders of amino acid metabolism (including organic acidurias), which can be identified by standard metabolic investigations, including plasma amino acid and urine organic acid analysis. Enzymatic deficiencies in amino acid metabolism result in the accumulation of toxic abnormal organic acid metabolites in the body and subsequent organ damage, with the brain being frequently affected. Vitamins are often core elements in amino acid degradation pathways and organic acid metabolism. The severity of symptoms and response to therapy may depend on the extent of the underlying enzyme deficiency.

The more common “OADs” include maple syrup urine disease (MSUD), isovaleric aciduria (IVA), propionic aciduria (PA), methylmalonic aciduria (MMA), and glutaric aciduria type 1 (GA1) [2,3]. The overall prevalence of these conditions, according to the Orphanet database (www.orpha.net (accessed on 1 February 2023)), varies, estimated as 1/100,000 for MSUD, 1–9/100,000 for IVA, 1–9/1,000,000 for PA, 1–9/100,000 for MMA, and 1/100,000 for GA1 [4] (Table 1). There are increasing numbers of pregnancies reported in females with OAD. This is reflective of the improvements in the management of these conditions, supported by the expansion of newborn screening [5,6]. To date, there are no reports of increased infertility in females previously diagnosed with OAD receiving effective treatment.

**Figure 1:**
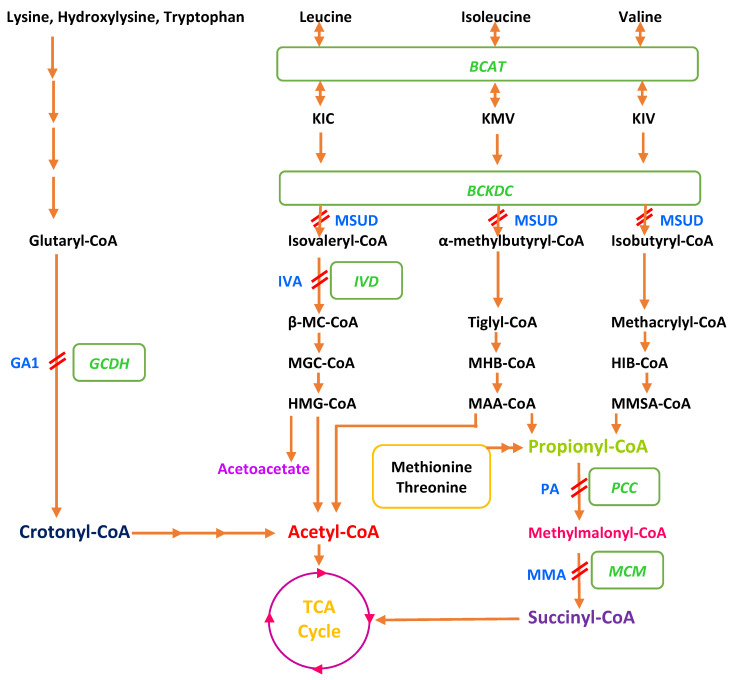
Main catabolic pathways of amino acids involved in organic aciduria. Abbreviations: GCDH: Glutaryl-CoA dehydrogenase, BCKDC: Branched-chain α-keto acid dehydrogenase complex, IVD: Isovaleryl-CoA dehydrogenase, PCC: Propionyl-CoA carboxylase, MCM: Methylmalonyl-CoA mutase, β-MC CoA: β-methylcrotonyl CoA, MGC CoA: β-methylglutaconyl CoA, HMG CoA: β-hydroxy-β-methylglutaryl CoA, MHB CoA: α-methylβ-hydroxyisobutyryl CoA, MAA CoA: α-methylacetoacetyl CoA, HIB CoA: β-hydroxyisobutyryl CoA, MMSA: methylmalonate semialdehyde, TCA: tricarboxylic acid, MSUD: Maple syrup urine disease, PA: Propionic aciduria, MMA: Methylmalonic aciduria, IVA: Isovaleric aciduria (IVA), GA1: Glutaric aciduria type 1, KIC: 2-keto-isocaproate/4-methyl-2-oxopentanoic acid, KMV: α-keto-β-methylvaleric acid/3-methyl-2-oxopentanoate, KIV: 2-keto-isovalerate/3-methyl-2-oxobutanoic acid, BCAT: branched-chain aminotransferase.

MSUD is characterized by the deficiency of an enzyme, branched-chain α-ketoacid dehydrogenase complex (BCKDC or BCKDHC), in the catabolic pathway of the branched-chain amino acids leucine, isoleucine, and valine. Propionic aciduria (PA) and methylmalonic aciduria (MMA) are characterized by the accumulation of propionic acid and/or methylmalonic acid due to the deficiency of propionyl-CoA carboxylase (PCC) or methylmalonyl-CoA mutase (MCM). PCC is a biotin-dependent mitochondrial enzyme that catalyzes the carboxylation of propionyl-CoA to methylmalonyl-CoA [17]. MCM catalyzes the reversible isomerization of L-methylmalonyl-CoA to succinyl-CoA using adenosylcobalamin (AdoCbl) as a cofactor [18]. Other causes of MMA are a defect in the transport or synthesis of MCM cofactor, adenosyl-cobalamin (AdoCbl), or deficiency of the enzyme methylmalonyl-CoA epimerase [19]. The enzymes deficient in PA and MMA have an indispensable role in the breakdown of the branched-chain amino acids valine, isoleucine, threonine and methionine. IVA is caused by the deficiency of the enzyme isovaleryl-CoA dehydrogenase responsible for the dehydrogenation of isovaleryl-CoA to produce 3-methylcrotonyl-CoA, and is involved in the metabolism of leucine. GA1 is caused by a deficiency of the enzyme glutaryl-CoA dehydrogenase which plays an important role in the catabolism of L-lysine, L-hydroxylysine and L-tryptophan [20] (Figure 1).

The clinical signs and symptoms of OADs vary between different disorders. Depending on the degree of enzymatic deficiency, these may present as intoxication or ‘metabolic encephalopathy’ in the newborn with cerebral oedema, coma and multi-organ failure, or as chronic intermittent presentations, with symptoms such as recurrent acidosis, lethargy, hypotonia, ataxia, neurological signs and seizures. Chronic progressive presentations may also be associated with failure to thrive, vomiting, developmental delay/regression, hepatomegaly, respiratory distress, cardiac dysfunction, osteoporosis, and recurrent infections.

The most important diagnostic investigation is organic acid analysis of urine by gas chromatography–mass spectrometry, followed by enzymatic assays and specific gene analysis [13,21,22,23]. Diagnostic confirmation is achieved by genetic testing.

The common aim of management in each of these conditions is to prevent catabolism by providing sufficient calories and essential amino acids to sustain metabolism and the correction of metabolic acidosis and hyperammonaemia [24]. During decompensation, patients with OADs are prone to metabolic organic acid intoxications, which may result in encephalopathy. The initial measures to prevent or correct metabolic decompensation include restricting intact or natural protein from the diet whilst supplementing with a precursor-free synthetic amino acid formula, where appropriate; the provision of energy either via enteral or parenteral means (intravenous dextrose or lipid); and the use of carnitine to enhance the excretion of toxic metabolites. The treatment aims to correct metabolic acidosis, hyperammonaemia, hypoglycaemia, and electrolyte abnormalities. Associated illnesses (e.g., infections, vomiting, and diarrhoea) also are treated.

In all OADs, commercially prepared low-protein foods and drinks are often necessary to achieve energy requirements and provide variety in the diet, particularly where natural protein is significantly restricted. This, along with ensuring an adequate provision of vitamins, minerals and essential fatty acids required according to age with avoidance of prolonged fasting, is essential. Prompt treatment of inter-current illness with appropriate medical treatment and the use of an emergency management plan to avoid/ameliorate catabolism remain the cornerstone of metabolic management.

An increasing number of women with rare IMDs are achieving healthy pregnancies [5]. Pregnancy represents a challenging biological stressor in patients with OADs. However, the reports of successful pregnancies in women with OAD continue to grow where once this may have been contraindicated. Case reports provide valuable insight into the implementation of dietary therapies during pregnancy and delivery management strategies.

In this overview, we summarise the experience of pregnancy in patients with organic acidurias, with emphasis on treatment strategies used to help inform practice in this area.

## 2. Methods

Data collection and interpretation in this review integrated a hybrid methodology combining meta-narrative and realist approaches [25,26,27]. All data and references were extracted from the PubMed engine, which accessed the MEDLINE database. Keywords used for the data search were: organic aciduria, maple syrup urine disease, propionic aciduria, methylmalonic aciduria, isovaleric aciduria, GA1, and pregnancy. For the purpose of this type of review on a subclass of IMDs, no inclusion or exclusion criteria were set. The Helsinki Declaration on Ethical Standards for Medical Research Involving Human Subjects was followed while conducting this evaluation of the literature, especially paragraphs 12 and 25, which govern the gathering and analysis of human data and call for a thorough understanding of the scientific literature.

## 3. Results (Background Review)

The search for ‘Organic Aciduria’ and ‘Pregnancy’ returned 44 results. The search for ‘Maple Syrup Urine Disease’ and ‘Pregnancy’ returned 90 results. The search for ‘Propionic Aciduria’ and ‘Pregnancy’ returned 42 results. The search for ‘Methylmalonic Aciduria’ and ‘Pregnancy’ returned 108 results. The search for ‘Isovaleric Aciduria’ and ‘Pregnancy’ returned four results. The search for ‘Glutaric Aciduria Type 1′ and ‘Pregnancy’ returned 25 results. To capture all reported cases of OADs and pregnancy, we also searched ‘Inherited Metabolic Disorders’, ‘Organic Acidemia’, ‘Isovaleric Acidemia’, and ‘Pregnancy’. The search returned 1417, 45, and 14 articles, respectively. Thirty-nine articles were pregnancy case reports in OADs [28,29,30,31,32,33,34,35,36,37,38,39,40,41,42,43,44,45,46,47,48,49,50,51,52,53,54,55,56,57,58,59,60,61]. Some of these articles included a number of pregnancy case reports; all successful pregnancies are discussed and detailed in Table 2.

## 4. Energy Balance and Caloric Adaptations to the Increased Demands of Pregnancy: Metabolic Considerations for Patients with OADs

Pregnancy is a dynamic state that involves profound anatomical, biochemical and physiological changes with considerable physiological and metabolic adaptations to meet the evolving caloric demands and growth of the foetus. These changes are driven by the increased physical and metabolic demands of pregnancy. The foetal demands increase with the progression of pregnancy. The energy requirement of basal metabolism is influenced by maternal prenatal nutrition and by foetal size [62]. Protein requirements are increased from early pregnancy with incremental changes during the course of the pregnancy to the time of delivery [63,64,65,66].

The overall anabolic phase occurs in the first two trimesters of human gestation with enhanced insulin sensitivity and increased maternal fat and fat-free mass [6,67]. The catabolic phase occurs in the third trimester, which is characterized by an accelerated breakdown of fat deposits. The glucose transporter (GLUT1) is considered to be the primary glucose transporter in the human placenta. Expression of GLUT1 increases during pregnancy [68,69]. Amino acids are transported across the placenta to support foetal growth, with an increased maternal–foetal gradient. There is a gradual increase in protein requirements during pregnancy, while maternal plasma amino acid levels are subsequently progressively decreased [70]. Protein deposition in maternal and foetal tissues increases throughout pregnancy, with most occurring during the third trimester [63]. From around 30 weeks gestation, placental hormones and adipocytokines drive increasing insulin resistance, favouring maternal catabolism and releasing glucose, fatty acids, and amino acids to meet increased foetal growth demands [69].

For the OADs, the first trimester of pregnancy may be particularly challenging. Metabolic decompensation may occur with nausea, poor appetite, ‘hyperemesis gravidarum’, and inter-current illnesses, making it difficult to achieve an adequate intake of calories and essential supplements. Labour and delivery are times of increased energy and protein requirements [71,72]. The postpartum period is the third high-risk period. It is a time of catabolism with the involution of the uterus and the breakdown of protein, associated with additional metabolic stress. Excess amino acids generated during this period can increase nitrogen load and could theoretically precipitate metabolic decompensation in a pregnant patient with an OAD [45,73]. In terms of teratogenic risks, there is no evidence that OADs influence the selection of regularly used antiemetics or vitamin supplements during pregnancy. Although there have been a limited number of cases, the administration of amino acid supplements and carnitine during pregnancy has not been reported to be teratogenic or to cause significant side effects.

Pregnancy presents a challenge to both the patient and the multidisciplinary team when trying to achieve the main aims of treatment. There is limited information about target metabolite biochemical ranges in pregnancy for patients with OADs and overall specific amino acid requirements during the different stages of pregnancy [56]. There is also no specific guidance on frequency for review and/or metabolic and obstetric care plans. Therefore, close monitoring of biochemical profiles, clinical judgement and treatment of catabolic episodes, in particular during the first trimester and immediate postpartum period, is required to prevent and to pre-emptively manage decompensation.

Breastfeeding of the neonate has been reported in affected women with OADs, with most cases being described in women with MSUD and MMA [74]. The benefits of breastfeeding are manifold, including the reduced risk of sudden infant death, allergic diseases, asthma, obesity, and type 2 diabetes [75]. Therefore, breastfeeding should be actively promoted and supported by the metabolic team. They will be best placed to provide dietetic support for successful and safe breastfeeding. Specific diet and monitoring guidelines to support breastfeeding currently exist only for MSUD. These guidelines recommend dietary monitoring and adjustment to support the extra energy and protein demands of lactation [74]. Insufficient caloric intake at this time could be a risk factor for metabolic decompensation, and this should be closely monitored.

## 5. Management of MSUD

A leucine-restricted diet is recommended, achieved by limiting natural protein intake. The total protein requirements are achieved by supplementing with a Branched Chain Amino Acids (BCAA) free synthetic amino acid supplement. Isoleucine (Iso) and valine (Val) supplements may be given to achieve appropriate target blood levels. A trial of thiamine supplementation should be documented (usually pre-pregnancy) [74,76]. Carnitine depletion is not a recognised feature in MSUD [34].

## 6. MSUD and Pregnancy

The main goals of treatment of MSUD during pregnancy are to increase protein intake to support foetal growth while maintaining BCAA levels within acceptable treatment targets [74]. This may require a combination of increased natural and BCAA-free synthetic amino acid, depending on blood levels. Energy intake must also support increased needs associated with pregnancy. Vitamin and mineral supplementation may be required depending on dietary and biochemical assessments, routinely carried out by a metabolic dietitian. Maintaining adequate caloric intake during pregnancy to help meet the additional energy demands of pregnancy is important, as is the need to ensure adequate energy provision during particularly vulnerable periods, such as labour, delivery and the early postpartum period. This can be achieved by infusion of intravenous dextrose +/− intravenous lipids [31].

The first three pregnancies in women with MSUD were reported in 1992 and 1998 (Table 2). Two of these were successful [34,35], and the third reported a maternal death at day 51 postpartum [35,36]. A favourable outcome of a pregnancy in a 22-year-old Turkish woman (who had only 2% residual branched-chain oxo acid dehydrogenase activity) was reported in 1998. The target plasma levels of the branched-chain amino acids were achieved (between 100 and 300 μmol/L). The patient’s leucine tolerance increased progressively from the 22nd week of gestation from 350 to 2100 mg/day [34].

A further two cases of successful pregnancies were documented in 2013 [31]. This patient was managed with a low-protein diet and branched-chain amino acid–free supplements. The leucine levels were persistently 500–1000 μmol/L. The branched-chain amino-acid-free supplements intake was increased from 60 g to 75 g at 15 weeks’ gestation. There was no natural protein intake on day 1 postpartum. This was gradually increased by 5 g increments to the usual pre-pregnancy intake of approximately 20 g over 1 week.

The other cases of pregnancy with severe MSUD were reported in 2015. In this case, the natural protein requirement began to increase by the fourth month of pregnancy. The protein and leucine intake (tolerance) peaked during the eighth month of the pregnancy. The mother resumed her pre-pregnancy diet with around 500 mg of leucine per day in the first few days following birth [30].

A report of pregnancy for an intermediate variant of MSUD was reported in 2018. The prior protein tolerance was 30 g of natural protein per day. The exchanges were increased to 60 g of natural protein by the third trimester and supplemented by synthetic proteins [29].

A further favourable outcome of pregnancy in a case of classical MSUD was reported in 2018. The BCAAs levels were maintained at: Leucine 100–300 μmol/L, isoleucine 100–300 μmol/L and valine 200–400 μmol/L. The patient’s protein tolerance increased significantly from the second trimester up to 27 mg/kg/d of leucine per day prior to delivery. The leucine intake was reduced to 200 mg/day on the day of delivery [77].

The treatment of an adolescent patient with intermittent MSUD and the resulting positive pregnancy outcome was described in 2021. This patient attended the metabolic clinic at 31 weeks gestation. The patient’s protein intake was approximately 30 g/day. Delivery was by emergency caesarean section with intravenous lipids and fluid supplementation [28]. Another similar case was reported from Japan in a thirty-one-year-old individual [37].

Each of these reports describe similar management strategies in terms of biochemical monitoring and dietary manipulation during pregnancy, labour, delivery, and the postpartum period. Each case described increasing natural protein tolerance during pregnancy, the provision of intravenous dextrose and lipids during labour and delivery, and a reduction in natural protein intake initially postpartum [32].

## 7. Management of PA and MMA

The proposed guidelines for the management of MMA and PA recommends regular monitoring of quantitative plasma amino acids, methylmalonic acid in plasma and urine, and acylcarnitine profile in dried blood or plasma [8]. L-carnitine at a dose of 100 mg/kg/day is recommended to maintain normal carnitine and CoA levels. A trial of parenteral B12 is recommended (ideally pre-pregnancy) to assess responsiveness in suspected cases. Some MMA patients may benefit from cobalamin treatment; however, biotin treatment for PA patients is not recommended [8]. Expert opinions advise against the primary use of sodium phenylbutyrate as an ammonia scavenger in MMA and PA during acute metabolic decompensation [8,10]. There is still limited evidence on the long-term efficacy of carglumic acid in MMA [78]. Recent evidence suggests that taking carglumic acid in addition to standard treatment may significantly reduce the number of emergency admissions related to hyperammonaemia in patients with PA and MMA [79].

In MMA and PA, natural protein restriction is advised to reduce the intake of propiogenic amino acids (valine, methionine, isoleucine, threonine). However, intake should not be over-restricted, as this may result in deficiencies in these essential amino acids, which are required to meet the needs for growth and anabolism. Synthetic amino acid formula may be needed to ensure an adequate intake of the unaffected amino acids where natural protein intake cannot be increased. Metronidazole (to reduce bacterial propionate production) may be considered. Acute management requires the temporary cessation or reduction in natural protein [24,80]. Acute illness management should include increased provision of energy via oral, enteral, or parenteral route as tolerated. MMA patients may experience late-onset disease complications such as chronic renal failure, chronic pancreatitis, and osteopenia [8,46,81].

## 8. PA/MMA and Pregnancy

Eight successful pregnancies have been described in six women with PA [35,51,82] (Table 3)**.** In the first documented case of pregnancy in PA, a pregnancy was reported at 6 weeks of gestation. The patient was treated with protein restriction (to 0.8 g/kg) and a propionic aciduria amino acid supplement formula for additional protein. L-carnitine (30 mg/kg) was prescribed [35]. There were no metabolic problems reported for these cases [38]. In general, a protein-restricted diet and carnitine supplementation were successfully employed to manage pregnancy in PA [35,38,51,52,53]. Complications during pregnancy included growth retardation and preeclampsia [51].

In the literature, seventeen successful pregnancies were described in 14 women with MMA, B12- responsive and non-responsive forms (Table 4) [35,51,82]. The first case report of a patient with MMA who carried a pregnancy to term was reported in 1995 [48]. Complications during pregnancy included hyperammonaemia [51], nausea and vomiting [40,73], hyperglycaemia, anaemia [40,46,47], proteinuria [46], and carnitine deficiency [47]. One infant was noted to have poor foetal growth during the pregnancy with documented poor nutritional baseline prior to conception [38]. One patient had three successful pregnancy outcomes, with complications only in the last pregnancy when she developed acute stress in labour due to possible placental abruption and preeclampsia [73].

There is a report of a woman affected with MMA who delivered two subsequent children who were also affected with MMA. The parents were unrelated. On day 5 of life, the first neonate presented with lethargy, hypothermia, and hyperbilirubinemia. Gas chromatography of a 24 h urine sample showed a high excretion of MMA, and B12-responsive MMA was confirmed on cultured fibroblasts. The mother had a subsequent successful affected pregnancy three years later. [49].

The protein restrictions for these case reports were described with increasing natural protein tolerance as the pregnancy advanced [40,42,46,50,73]. Precursor-free amino acids were utilised in two cases where insufficient natural protein intake was tolerated [38,40]. In one reported case, the addition of nocturnal corn starch was used dosing during pregnancy to prevent a fasting state and reduce the catabolism of odd-chain fatty acids [46]. The majority of cases were treated with vitamin B12 and carnitine, often given in higher doses and adjusted with biochemical metabolite monitoring. Nine pregnancies were carried to term. The most common method of delivery was via C-section. In almost all cases, intravenous dextrose was used to reduce the risk of metabolic decompensation. Intravenous carnitine was given during the delivery period in three cases [40,47,73]. There were no long-term complications for the MMA patients in the peripartum or postpartum periods. One case described a successful breastfeeding experience which was supported by a protein intake of up to 1.68 g/kg in addition to extra calories [40]. Cardiac complications and occult cardiomyopathies have been reported in PA patients [83,84]. Metabolic strokes and associated neurologic sequelae, particularly during periods of catabolism, are also reported [82]. Cardiac assessment is recommended at the early stages of pregnancy and during pregnancy [51].

## 9. Management of IVA

The aim of treatment is to maintain a state of anabolism, reducing the formation of isovaleryl-CoA formation from leucine catabolism. Natural protein restriction is recommended to reduce the isovaleric acid load. Leucine intake should supply the recommended levels of intake. It is recommended to prescribe L-carnitine to maintain an adequate free carnitine level in blood and to add L-glycine in severe types [85,86]. Detoxification of excess isovaleric acid is achieved by conjugation with glycine, hydroxylation, and excretion in urine as 3-hydroxyisovaleric acid. The absence of 3-hydroxyisovaleric acid in the urine suggests metabolic stability [54]

## 10. IVA and Pregnancy

Of note, there are no consensus guidelines for the dietary management of pregnancy in IVA. A number of cases of pregnancies in IVA have been described from 1984, with eight successful pregnancies (including one twin pregnancy) (see Table 5) [54,55,56,57]. Treatments included leucine-free formula, protein-restricted diet, l-carnitine or glycine supplementation or a combination thereof, which are comparable to non-pregnancy IVA management strategies. Information on dietary prescriptions used in pregnancy was limited. Interventions required during pregnancies included increased glycine and L-carnitine supplementation in two pregnancies and increased protein intake in the latter part of the pregnancy in another case. Symptom management of malodour from urine in trimester 2 in one pregnancy was attributed to an increase in milk consumption and improved with milk cessation. Protein intakes, in this case, varied hugely, with reported intakes from 32 to 108 g per day. Isovalerylglycine was the only abnormal metabolic detected in the urine at 20, 31 and 36 weeks gestation [54]. Hyperemesis gravidarum was reported in three cases and was managed with intravenous glucose L-carnitine and oral glycine. [55,56,57]. Two of these pregnancies required significant increases in carnitine and glycine doses to manage low levels. Successful pregnancy outcomes were reported in all cases [54,55,56,57].

## 11. Management of GA1

Dietary treatment of GA1 varies according to the age of diagnosis and symptom severity. The aim of the treatment is to limit dietary lysine, the most quantitatively relevant amino acid precursor of the neurotoxic glutaric acid and 3-hydroxyglutaric acids [87], while maintaining sufficient intake of protein, energy and essential nutrients to meet requirements [17]. Carnitine supplementation is associated with risk and mortality reduction [88]. Hence, lifelong carnitine supplementation is recommended [89,90]. The recommended dose is 100 mg carnitine/kg/day, to be adjusted to maintain plasma carnitine concentration within the normal range [14]. The international consensus guidelines recommend a relaxation of lysine restriction after six years of age; however, many centres may recommend acute management of intercurrent illness or surgery with increased dextrose intake. The suggested emergency treatment (pre-pregnancy) includes stopping natural protein intake for 24–48 h and supplementing with intravenous dextrose and L-carnitine [59].

## 12. GA1 and Pregnancy

There are no consensus guidelines for dietary management during pregnancy or breastfeeding. The physiological changes of pregnancy and the catabolism associated with labour and delivery can impose the risk of a neurological ‘crisis’ in GA1 [58]. Therefore, the vulnerable stages of pregnancy may also necessitate this treatment to minimise catabolic stress and prevent neurological crises. This can be achieved by adequate caloric intake such as IV dextrose and lipid in addition to carnitine supplementation. For most documented reports on GA1 in pregnancy, the patients were mostly asymptomatic or showed only mild neurologic symptoms.

There are a number of successful pregnancies reported in GA1 [58,60] (Table 6). The outcomes of three pregnancies involving two women who had undiagnosed GA 1 were reported in 2007. Case 1 had a normal pregnancy and was delivered at term. This woman had a previously uneventful pregnancy and delivery. In Case 3, a child was born to a woman who had GA1 following a normal pregnancy and delivery. Although untreated and not supplemented with carnitine, both women had no metabolic decompensation during gestation or in the postpartum period [60]. In another report in 2008, a further two women, the first in her second pregnancy and the second in her third pregnancy, had normal pregnancies, deliveries and healthy newborns. The diagnosis of GA1 in both women was only established following a positive newborn screening test in their babies [61]. To our knowledge, there are five pregnancies reported in four women diagnosed with GA1 [38,59,60]. A 23-year-old primigravid woman with a history of GA1 presented for a scheduled caesarean section at 36 weeks of gestation. Preconception, she was treated with a low protein diet (40 g/d), L-carnitine, and riboflavin supplements. Carnitine supplement was increased from 0.5 g to 2 g daily at 18 weeks of gestation. The delivery management was pre-planned with a detailed emergency C-section protocol (40). Limited information is available on treatments used, and there is no information on dietary treatments employed.

In our practice, we followed a female patient with GA1 who had two clinically uneventful pregnancies. Although asymptomatic at diagnosis (age 11 years), dietary treatment was commenced with restriction of natural protein (tryptophan and lysine) and synthetic protein substitutes were prescribed to meet daily protein and micronutrient requirements according to the Irish practice [91]. In both pregnancies, there were slight increases in natural protein exchanges, with increasing protein requirements across the trimesters achieved with higher synthetic protein intake. Additional energy was provided with glucose polymers, fat supplements, and sugar-based beverages. Carnitine and essential fatty acid supplementation continued with the addition of vitamin and mineral supplementation. The weight gain was acceptable for both pregnancies, and the risk of acute decompensation was proactively managed. Catabolic stress during labour and delivery was mitigated by providing effective pain relief, adequate hydration, calories, and the maintenance of acid–base balance pre-, during, and post-delivery.

## 13. Discussion

In this overview, we summarise the metabolic experiences of pregnancy in patients with organic acidurias, with emphasis on treatment strategies used in all stages of pregnancy and the postpartum period. A summary of the reports reviewed with interventions is provided in Table 2. The overall summary of reports (in the absence of pregnancy-related clinical practice guidelines) suggests that catabolism should be prevented or minimized in all stages of pregnancy and the postpartum period with OADs using intensive dietary interventions (enteral or parenteral nutrition when needed) [34,35].

Overall the foetal outcomes were favourable in all the OADs described [31]. However, although there is an increase in reported successful pregnancies in this reported cohort, there is a paucity of data on long-term outcomes for the offspring. In the case of MMA, favourable outcomes were achieved despite high levels of methylmalonic acid in the serum and urine, which may suggest that elevated levels of MMA may not be teratogenic [49]. Additionally, it has been suggested that there may be foetal metabolism of MMA, as a reduction in MMA levels was shown in one case report [46]. Poor foetal development was reported in one case that was most likely caused by insufficient dietary intake [38].

Amino acids cross the placenta by an active transport mechanism. Leucine can rapidly cross the placenta. It has been estimated that toward the latter part of gestation, 90% of foetal plasma leucine is derived from maternal circulation. The exposure of the foetus to a high concentration of leucine might have a negative impact on its growth and development [92]. Similarly, abnormally high maternal organic acid metabolites may potentially cross the placenta and negatively impact foetus development. Therefore, careful monitoring of the mother’s metabolic status during pregnancy is essential to minimize potential risks to the foetus. Other complications reported in maternal phenylketonuria (PKU), including congenital heart defects, microcephaly was not reported in this series [93]. In addition to dietary intervention, a number of adjuvant treatments were described.

L-carnitine has an important role in the management of OADs. Carnitine is an important molecule contributing to energy production and the metabolism of fatty acids [94]. It mediates the transport of fatty acids into the mitochondria. It possesses antioxidant properties that might have a role in protecting against the oxidative stress promoted by BCAA [95,96]. L-carnitine may also protect against lipid peroxidation [97]. L-carnitine might have an important role in foetal growth [98,99]. It has been reported that low carnitine levels may negatively influence foetal maturation [100,101]. Carnitine deficiency can lead to muscle weakness and cardiomyopathy [102].

It has been reported that L-carnitine levels are lower in MSUD patients compared with the general population. Studies have shown that oxidative stress may be involved in the neuropathology of MSUD. In vitro studies demonstrated that leucine and α-ketoisocaproic acid may cause DNA damage. In these studies, L-carnitine was able to significantly prevent DNA damage [103]. It has also been reported that L-carnitine supplements may enhance the formation and excretion of short-chain acylcarnitines in PA [104]. The current clinical practice guideline suggests that L-carnitine is useful in the management of patients with MMA and PA [24]. Carnitine supplement to MMA patient can increase the urinary excretion of hippurate and short-chain acylcarnitines, and reduces the excretion of methylmalonate and methylcitrate [105]. Patients with IVA may also have low plasma levels of free carnitine. Studies have shown that L-carnitine conjugated isovaleric acid earlier than glycine. It has been suggested that supplementation with L-carnitine might enhance the excretion of isovalerylcarnitine and reduce or prevent further hospitalizations [85,86].

L-carnitine supplementation also enables physiological clearance of glutaryl-CoA by conjugation with carnitine. In GA1 patients, supplementation with L-carnitine resulted in beneficial effects by reducing levels of toxic intermediate metabolites [106]. Notwithstanding the beneficial effect of L-carnitine therapy, there is still no consensus on the dose and duration of treatment. Furthermore, there are no controlled trials on its safety when high doses are used or when it is prescribed for longer periods.

Another adjuvant therapy is the intermitted use of oral antibiotics (such as metronidazole) to decrease propiogenic anaerobic gut bacteria in MMA and PA [24].

The development of clinical guidelines can directly improve management, bring further insights, and advance research. Guidelines for the management of pregnancies in OADs are limited, consistent with the limited experience to date [107]. Clinicians should be aware of potential complications and carefully consider how best to manage these conditions during pregnancy. Preplanning pregnancy should include consideration of potential complications and detailed monitoring plans throughout the trimesters. Avoidance of catabolism throughout pregnancy, labour, and the postpartum period is very important. This can be achieved by providing adequate caloric, protein, and micronutrient intake in conjunction with close monitoring of metabolic and nutritional status. Patients should be closely followed up by a specialised metabolic and dietetic team, which can implement a planned approach to conception and pregnancy. Close liaison with other specialists, in particular, perinatal specialists, is required for optimal outcomes. In this context, with the increasing number of these high-risk pregnancies expected, increased education in this discipline is required.

Finally, consideration should be given to breastfeeding, and patients should be counselled accordingly [74]. Mothers who have OADs should be encouraged to breastfeed. However, it is important to closely monitor the mother’s metabolic control with supplemental essential amino acids and to monitor the infant’s nutritional status, growth, and development.

## 14. Conclusions

The development of shared care guidelines and outcome monitoring of the offspring will aid in continued successful pregnancy outcomes for women with OADs. Further research is required to develop recommendations for amino acid precursor essential requirements at the different stages of the pregnancies, the development of novel predictive biomarkers for early detection of decompensation, and to monitor therapies. Developing new targeted therapies and rescue medication to effectively prevent and treat acute decompensation is another important area for further research.

## Figures and Tables

**Table 1:** Classical Organic Aciduria- Clinical and Biochemical features.

OADs	OMIM	Gene Symbol	Salient Clinical Features	Salient Blood Features	Salient Urine Features	Biochemical Monitoring
MSUD	248600	*BCKDHA*, *BCKDHB* and *DBT*	Encephalopathy, Developmental Delay, Intellectual disability	Elevation of leucine, isoleucine, and valine, alloisoleucine present	Elevated: 2-oxoisocaproate, 2-oxo-3-methylvalerate, 2-oxoisovalerate, 2-hydroxyisovalerate, 2-hydroxyisocaproate, 2-hydroxy-3-methylvalerate	Plasma BCAA
PA	606054	*PCCA* or *PCCB*	Encephalopathy, Developmental Delay, Intellectual disability Seizures, Basal ganglia lesions Optic atrophy, hearing loss, pancreatitis, cardiomyopathy, Growth retardation anaemia, leukopenia, immune deficiency Renal failure [7].	Elevated glycine, low glutamine, normal methionine, elevated propionylcarnitine (C3), presence of 2-methylcitrate [8,9]	Presence of 3-hydroxypropionate, 2-methylcitrate, tiglylglycine propionylglycine, lactic acid, absence of methylmalonic acid	Regular monitoring of serum ammonia, [10]
MMA	251000	*MUT*	Encephalopathy, Developmental Delay, Intellectual disability Seizures, Basal ganglia lesions Optic atrophy, hearing loss, pancreatitis, cardiomyopathy, Growth retardation Renal failure	Ketoacidosis hyperammonaemia, hyperglycinaemia. Pancytopenia. Elevations of methylmalonic acid, 3-hydroxypropionate, and presence of 2-methylcitrate [8].	Ketonuria, Elevated levels MMA and the presence of 3-hydroxypropionate, 2-methylcitrate, and tiglylglycine [11]	Plasma amino acids, plasma and urine methylmalonic acid levels, serum acylcarnitine profile and free and total carnitine levels [11]
IVA	607036	*IVD*	Metabolic acidosis, Encephalopathy, Developmental Delay, Intellectual disability neutropenia [12]	Metabolic acidosis (with elevated anion gap), elevated lactate, hyperammonaemia	Increased excretion of 3-hydroxybutyric acid and 3-hydroxy-isovaleric acid [13]	Amino acids and carnitine in plasma, urinary isovalerylglycine and plasma isovalerylcarnitine levels
*GA1 GCDH*	231670	*GCDH*	Progressive macrocephaly, acute encephalopathic crisis, basal ganglia injury, nonspecific neurologic abnormalities Developmental delay/ Intellectual disability	Elevated glutaric acid, 3-hydroxyglutaric acid, glutaconic acid, and glutarylcarnitine [14]	High plasma glutaryl carnitine [15]	Quantitative analysis of plasma amino acids [16]

Abbreviations: MSUD: Maple syrup urine disease; PA: Propionic aciduria; MMA: Methylmalonic aciduria; IVA: Isovaleric aciduria (IVA); GA1: Glutaric aciduria type 1; BCAAs: Branched-chain amino acids.

**Table 2:** Published literature on pregnancies in MSUD.

Maternal Age (Years)	Management during Pregnancy	Timing of Delivery (Weeks)	Mode of Delivery/ Treatment in Labour	Birth Weight (g)	Foetal Outcome	Maternal Outcome	Ref
17	Protein-restricted diet. Recommended total protein is 80 g/day. Diet is adjusted once to twice weekly depending on the BCAA levels	37	CS Intralipid 20% (2 g/kg) + D10% were started during the peripartum period	3000	Normal	No metabolic problems	[28]
28	Initial diet of 30–40 g natural protein in the first and second trimester was increased to 60 g natural protein by the third trimester, supplemented by one MSUD	40	SVD	3740	Normal	No metabolic problems	[29]
31	Natural protein requirement continuously increased from the fourth month of gestation. The daily intake of natural protein was increased to 15 g. During the second half of pregnancy, a further increase to 30 g of natural protein was required. The maximal protein and leucine intake was in the eighth month of pregnancy	41	Dextrose IV infusion (220 g/24 h) continued for the next 2 days.	3430	Normal	No metabolic problems	[30]
Unknown	Low-protein diet with increases in allowances over the duration of the pregnancy according to amino acid levels	40	IV Dextrose 20% IV Intralipid 20%	3336	Normal	No metabolic problems	[31]
21	Unknown	41	CS Natural protein intake was zero on day 1 and increased to 9 g daily over 3 days	3405	Normal	No metabolic problems	
31	Natural protein from diet plus synthetic protein. Isoleucine and valine supplements	33 w 4 d	CS IV Dextrose 20% IV Intralipid 20%	1760	Normal	No metabolic problems	[32]
28	In early pregnancy, 87% of the protein was provided by BCAA-free medical formula. Late in pregnancy, the BCAA-free formula comprised 70% of protein	37	CS Parenteral nutrition continued until patient was able to take at least 50% of goal calories PO for 3 days	2740	Normal	No metabolic problems	[33] ‡
22	BCAA tolerance increased progressively from around 21 weeks of gestation. During the second half of pregnancy, leucine intake was gradually increased to 2100 mg/day.	36	SVD	2860	Normal	No metabolic problems	[34]
25	Whole protein restriction at 0.6 g/kg plus continued use of BCAA-deficient formula providing 0.6 g/kg protein equivalents. Through the T2, whole protein intake was increased to 0.8 g/kg. For the remainder of pregnancy, 1.5 g/kg whole protein	40	SVD IV Dextrose continued for 12 h after delivery	2600	Normal	No metabolic problems	[35] †
19	Controlled diet of 200 g per day of BCAA-free milk, 1.2 g/kg per day of protein, 1 g/kg per day of fat, and a total energy intake of 2500 kcal/day,	36 w 2 d	Unknown	2736	Cardio-pulmonary arrest	No metabolic problems	[36]
31	Total protein intake: Pre-pregnancy 43 g, T1: 45 g, T2: 49 g, T3: trimester 50 g, Perioperative period ~33 g, Postpartum 42 g.	37 w 4 d	CS Dextrose 10% 80 mL/h was started six hours before the surgery.	2673	Normal	No metabolic problems	[37] **

Abbreviations: SVD: spontaneous vaginal delivery. CS: caesarean section. IOL: induction of labour, T: Trimester, BF: Breast Feeding, PO: By mouth, *D10NS:* 10% dextrose in normal saline. † This patient was supplemented with carnitine, 50 mg/kg, at 28 weeks of gestation. Carnitine supplement was increased in increments to 200 mg/kg. ‡ For a low normal serum vitamin B12 concentration, this patient was supplemented with Vitamin B12. ** This patient was prescribed Vitamin B1, folic acid, Fe, zinc, selenium, and carnitine to support her nutritionally.

**Table 3:** Published literature on pregnancies in PA.

Maternal Age (Years)	Treatment during Pregnancy	Diet	Timing of Delivery (Weeks)	Mode of Delivery/ Treatment in Labour	Birth Weight (g)	Foetal Outcome	Maternal Outcome	Ref
22	L-carnitine 30 mg/kg with a gradual increase (30–100 mg/kg)	Whole-protein restriction to 0.8 g/kg body weight + PA protein formula equivalent of 0.5 g/kg.	37	SVD IV Dextrose	2500	Normal	No metabolic problems	[35] *
26	L-carnitine (no dose available)	Whole-protein restriction, medical formula,	36.5	IV Dextrose Van Calcar 2015/clinic experience	Unknown	Normal	No metabolic problems	[51] *
28	L-carnitine, Biotin	Whole-protein restriction, medical formula	31			Growth retardation	Preeclampsia No metabolic problems	[51] **
30	L-carnitine, Biotin	Whole-protein restriction, medical formula	32			Normal	Preeclampsia No metabolic problems	[51] **
21	Biotin (10 mg/d) + L-carnitine (50 mg/kg/d)	By 37 weeks gestation 10% increase in protein intake was recommended	37 w 3 d	SVD *D10NS* 150 mL/h L-carnitine: 80–90 mg/kg	3930	Normal	No metabolic problems	[51]
31	T1: Tyrosine (550 mg/day), Biotin 5 mg/day Carnitine 50 mg/kg/day T2: Tyrosine 400 mg/g), Biotin 5 mg/day, Carnitine 60 mg/kg/day T3: Tyrosine 200 mg/day, Biotin 5 mg/day, Carnitine 75 mg/kg/day Lactation: Tyrosine 150 mg/day, Biotin 5 mg/day, Carnitine 50 mg/kg/day	T1: Protein, g/kg/g 1.0/AA mixture 0.4 g/kg/day T2: 1.11/AA mixture 0.5 g/kg/day T3: 1.2/AA mixture 0.6 g/kg/day Lactation: 1.3/AA mixture 0.65 g/kg/day	37	CS IV Dextrose 10% + low dose of bicarbonate	2200	Normal	No metabolic problems	[52]
35	L-carnitine 1 g/day	Protein approximately 0.8 g/kg body mass/day,	31	CS	First twin weighed 1550 g, the second weighed 1340 g	Normal	No metabolic problems	[53]
26	L-carnitine B9	Unknown	40	SVD IV Dextrose	4410	Normal	No metabolic problems	[38]

Abbreviations: SVD: spontaneous vaginal delivery. CS: caesarean section. IOL: induction of labour, T: Trimester, BF: Breast Feeding, PO: By mouth, *D10NS:* 10% dextrose in normal saline. * Same patient. ** Same patient.

**Table 4:** Published literature on pregnancies in MMA.

Maternal Age (Years)	Metabolic Treatment during Pregnancy	Dietary Treatment During Pregnancy	Timing of Delivery (Weeks)	Mode of Delivery/ Treatment in Labour	Birth Weight (g)	Foetal Outcome	Maternal Metabolic Outcome	Ref
18	No vitamin B12, carnitine (no dose available)	64 g protein/day (preconception prescription)	38	CS	3288	Normal	Hospitalization and IV fluids twice at 24 weeks due to nausea, vomiting and lethargy	[37]
24	Vitamin B12, preconception dose (actual dose not available) Carnitine (no dose available)	Up to 45 g/day	42	CS	3714	Normal	None	[37] **
35	5 mg B12 every other day throughout pregnancy, delivery and labour 1500 mg L carnitine twice daily	45 g/day trimester 1 and 2 and 80 g/day trimester 3 (actual intake) 70 g/day)	32	CS L-carnitine 50 mg/kg IV 6 h for 24 h and then reduce to 25 mg/kg IV 6 h until stabilised	1459	Normal	Acute stress at labour due to possible placental abruption and preeclampsia	[37] **
29	No Vitamin B12 Carnitine Vitamin D, C and folic acid (no doses available)	47 g/day	39	SVD	3095	Normal	None	[37] **
24	B12, L-Carnitine	B12, B9, Iron/vitamin D/calcium supplements	38	SVD IV Dextrose	2850	Normal	Mild hyperammonaemia in pregnancy.	[38]
19	B12, L-Carnitine	Multivitamin supplement. Amino acid supplement. Inadequate nutrition—poor foetal growth	35	CS IV Dextrose	1530	Growth retardation	No metabolic problems	[38]
27	B12, L-Carnitine		40	SVD	3300	Normal	No metabolic problems	[39]
31	L-Carnitine T1: 3 g/day T2 + 3: 4 g/day BF: 4 g/day Isoleucine + Valine T1: 100 mg/day T2 + 3: 150 mg	Total Protein: T1:1 g/kg protein T3:1.2 g/kg protein BF 1.68 g/kg protein Supplements: Protein powder Dextrose polymer Precursor-free L-amino acids (only postpartum)	38	CS IV Dextrose 10% + Carnitine (Days: −1,0,1,2)	3280	Normal	No metabolic problems- T1,2: Nausea and vomiting in T1: Hyperglycaemia T3: Persistent anaemia	[40]
29	No B12	Unknown	37	CS	2480	Normal	No metabolic problems	[41]
24	Aspirin, folic, B12, L-Carnitine	Total protein intake was limited to 1.1 g /kg/d,	At term	Dextrose 10% with 0.9% saline	3940	Normal	No metabolic problems	[42]
23	B12 (1 mg, every other day, IM) L-Carnitine (1 g tds) Folic acid (5 mg tds) Betaine (1 g tds)	No protein restriction prior/during pregnancy/delivery	40	Unknown	3300	Normal	No metabolic problems	[43] †
34	1 mg B12 every 3 days, low-molecular weight heparin	Not stated	39	Unknown	2420	Normal	No metabolic problems	[44] ‡
Unknown	B12 1 mg fortnightly. Increased to daily 1 mg B12 from delivery to day 6. Then, reduced to 1 mg B12 every second day with a gradual reduction to 1 mg fortnightly	Unknown	36	CS IV Dextrose 10% 10 mg/kg/min	Unknown	Normal	No metabolic problems	[45]
20	Metronidazole Bicarbonate Erythropoetin Iron L-Carnitine	Cornstarch 1 g/kg Protein: T1:40 g T2: 55 g	36 w 5 d	IOL IV Dextrose 20% IV Bicarbonate Stop protein until D2 postpartum	3220	Normal	Aneamia T2: Proteinuria No metabolic problems	[46]
24	IM OH-cobalamin 5 mg/week. Oral carnitine 2 g/day	Not stated	38	CS Dextrose and carnitine infusion	2940	Normal	12 w gestation: macrocytic anaemia plasma free carnitine deficiency No metabolic problems postpartum	[47]
23	No B12, T2: L-Carnitine started as levels low (dose unknown)	Energy: 81–130 kcal/kg/day Fat: 3.2–5.6 g/kg/day Carbohydrate: 10–16 g/kg /day Protein: 1.6–4.1 g/kg/day	Unknown	SVD	3500	Normal	No metabolic problems	[48] *
Unknown	Unknown, large dose B12 given during last 9 weeks gestation	Not stated	40	Unknown	2900	MMA child	No metabolic problems reported	[49] **
Unknown	B12 at week 27 of gestation	Not stated	41	SVD	2350	MMA child	No metabolic problems reported	[49] **
23	Cyanocobalamin 500 mg BD PO + L-carnitine 1 mmol/10 mL OD PO + Essential fatty acids supplement	Dietary protein restriction (not quantified), adequate carbohydrate supplementation	34	CS	1900	Normal	No metabolic problems	[50]

Abbreviations: SVD: spontaneous vaginal delivery. CS: caesarean section. IOL: induction of labour, T: Trimester, BF: Breast Feeding, PO: By mouth, *D10NS:* 10% dextrose in normal saline. * First reported case of pregnancy in a patient with MMA. ** Same patient. † This patient has combined methylmalonic aciduria and homocystinuria, cblC type. ‡ This patient has late-onset combined homocystinuria and methylmalonic aciduria.

**Table 5:** Published literature on pregnancies in IVA.

Maternal Age (Years)	Treatment during Pregnancy	Diet	Timing of Delivery (Weeks)	Mode of Delivery/ Treatment in Labour	Birthweight (g)	Foetal Outcome	Maternal Outcome	Ref
21	Iron (dose unknown)	Week 20–38, 24 h recall 32–108 g protein per day Urinary urea nitrogen to total nitrogen ratio = > 80 g protein per day	At term	Unknown	3700	Normal	In T2: Increased urine odour associated with increase in milk consumption disappeared with cessation of milk	[54] *
Pregnancy 1 at age 21 Pregnancy 2 unknown age Pregnancy 3 unknown age	Carnitine 2.5 g twice daily + glycine 4 g three times daily	Low protein diet (amount not available)	37	CS /IV Dextrose carnitine (100 mg/kg/24 h) + sodium benzoate (loading dose 250 mg/kg over 90 min followed by 250 mg/kg /24 h	3140	Normal	No metabolic problems	[55] †
	Carnitine 3 g twice daily	Protein-restricted diet (amount not available)	Unknown	Unknown/IV L carnitine 100 mg /kg/day + sodium benzoate 250 mg/kg and IV dextrose	2920	Normal	In month 2 hyperemesis gravidarum required management with antiemetics, IV dextrose. IV carnitine 100 mg/kg was required as oral declined	[56] †
	Same as pregnancy 2	Same as pregnancy 2	Unknown	Unknown/ same management as pregnancy2	3940	Normal	Same problem occurred at month 2 in this pregnancy	[56] †
Pregnancy 1 unknown age Pregnancy 2 unknown age	Carnitine (1320 mg × 3 times daily + glycine 6 g three times daily Month 5: glycine increased to 10 mg three times daily Month 6: 15 mg three times daily	Preconception: protein-restricted diet and 30 g leucine-free formula, no data available for during pregnancy	Term	Mode of delivery not available /IV Dextrose 10%; 2.5 L day and IV L carnitine 200 mg/kg/day and oral glycine 15 mg/day	3980	Normal	No metabolic problems	[56] ††
	Carnitine + glycine Required 100 g glycine per day and 10 g L carnitine per day	Leucine-free formula increased to 80 g per day	Term	Same as pregnancy 1	4200	Normal	No metabolic problems	[56] ††
25	Carnitine 9 g/day	Protein-restricted diet and leucine-free formula (amounts not available) In the last trimester protein intake was monitored and controlled (amounts not available)	Unknown	Unknown/ No additional medical care during labour	Unknown, Low to normal growth of foetuses compared to single pregnancies was observed	Favourable outcome for mother and twins. Two episodes of hyperemesis gravidarium in month 4 and 5 were treated with IV dextrose and 100 mg/kg L carnitine per day		[56]
20	Preconception doses used: Carnitine 70 mg/kg/day) + glycine 140 mg/kg/day	From week 5 gestation gradual adaption in diet to 40 kcal/kg/day and 1.5 g protein/kg/day. Multivitamin and iron supplementation (doses not available)	35	SVD	2718	Normal	No metabolic problems	[57]

Abbreviations: SVD: spontaneous vaginal delivery. CS: caesarean section. IOL: induction of labour, T: Trimester, BF: Breast Feeding, PO: By mouth, *D10NS:* 10% dextrose in normal saline. * First reported case. This patient has had two pregnancies. The first was terminated at 6 weeks’ gestation. † Same patient. †† Same patient.

**Table 6:** Published literature on pregnancies in GA1.

Maternal Age (Years)	Treatment during Pregnancy	Diet	Timing of Delivery (Weeks)	Mode of Delivery/ Treatment in Labour	Birth Weight (g)	Foetal Outcome	Maternal Outcome	Ref
21	B12, B2, Carnitine	Unknown	39	SVD	3017	Normal	No metabolic problems	[38]
18	B12, B2, Carnitine	Unknown	38	IOL IV Dextrose	4030	Normal	No metabolic problems	[38]
23	Carnitine 1500 mg bd	Natural protein (16–24 g/day), synthetic protein (60–68 g/day) (from 4 to 33 weeks of gestation)	38	CS IV Dextrose 10%, 12 g of natural protein + 68.3 g synthetic protein	3615	Normal	No metabolic problems	[58] †
28	Carnitine 1500 mg bd	Natural protein (20–25 g/day), synthetic protein (6068 g/day) (from 4 to 36 weeks of gestation)	38w6d	CS 2 days pre-delivery: reduced half natural protein Date of delivery: 0 g of natural proteins, +75 g synthetic protein IV Dextrose 10% Day 1 and 2 post-delivery: Half natural protein + 75 g synthetic protein	4470	Normal	No metabolic problems	[58] †
23	Carnitine supplement was increased from 0.5 g to 2 g daily at 18 weeks’ gestation	Pre-pregnancy protein 40–50 g/d continued during pregnancy	36	CS IV infusion of L-carnitine 667 mg in 10% dextrose at 125 mL/h. L-carnitine and 10% dextrose infusion was continued perioperatively until regular diet was resumed.	2680	Normal	No metabolic problems	[59]
24	Unknown	Unknown	Unknown	Unknown	3515	Normal	No metabolic problems	[60] ‡
20	Not supplemented with carnitine	Unknown	unknown	unknown	3160	Normal	No metabolic problems	[60]
Two women with GA1, were diagnosed only flowing testing their newborn children						Normal	No metabolic problems	[61]

Abbreviations: SVD: spontaneous vaginal delivery. CS: caesarean section. IOL: induction of labour, T: Trimester, BF: Breast Feeding, PO: By mouth, *D10NS:* 10% dextrose in normal saline. † Same patient. ‡ This patient had a previously uneventful pregnancy and delivery. She was diagnosed a few days following the detection of low free carnitine in the neonatal screening of her second baby.
